# General Rules in Operating with the Galvanic Cautery

**Published:** 1876-05

**Authors:** C. T. Deane


### GENERAL RULES IN OPERATING WITH THE GALVANIC CAUTERY.

By C. T. DEANE, M.D.

1. A good battery is indispensable.

2. Be sure, by a thorough examination *yourself*, that the battery and instrument are in proper order, and will work correctly, so as to cause no confusion or delay while the operation is in progress.

3. Be provided with one or more platinum wires besides the one connected with the instrument, for reasons stated in former article.

4. The contraction of the loop being controlled by the screw, should, in all cases, be slow and gradual, yet interrupted so as to insure a perfect cauterization of each stratum as it passes through. The speed with which the screw is turned is of great importance, so far as the prevention of hemorrhage is concerned, and the best rule to follow is, the greater the vascularity and liability to hemorrhage the slower the speed, and *vice versa*.

5. Towards the close of the operation, and as the circle of wire becomes smaller, the current of electricity should be proportionally decreased.

6. Traction on wire should be carefully avoided until it has passed well into the sub-mucous tissues.

7. The wire loop should never be brought to a white heat when passing through superficial tissues. The current is to be controlled and moderated so as to heat the wire red, this being more desirable as the tissues are more thoroughly cauterized, thus lessening the probabilities of hemorrhage. The cautery loop is to be introduced while cold, and made to embrace the cervix. The loop is then moderately tightened, and the electric circuit completed, little or no contraction of the loop being effected, so that the superficial tissues are thoroughly cauterized. Wnen the wire has entered the structure to a sufficient depth, firm and steady traction is made by means of a vulsellum or tenaclum and the intervening tissues slowly severed by further contraction of the loop. By such proceedings, the surface from which the parts are removed will present more or less of a concavity, and very similar in appearance to a raw potata cut in half by a rusty knife. There will not be the slightest hemorrhage whatever, and the operator, if he desires, may apply a simple dressing of cotton saturated with glycerine to the exposed surface. The after treatment does not differ from other operations of this nature—no opium being necessary, as there is no pain unless the sides of the labia may accidentally have been burned. Perfect rest should be enjoined for a few days, no motion of the bowels being allowed, and the urine taken away with a catheter. If an examination be made after a few days, the surface will be found to be covered with healthy granulations.

Out of a series of sixty cases demanding amputation of the cervix uteri operated on by Simon, five (5) terminated fatally. I have operated nine (9) times without losing a case.

